# Cardiorespiratory fitness and socioeconomic influences in Chilean schoolchildren: a cross-sectional study

**DOI:** 10.1016/j.jped.2024.06.010

**Published:** 2024-08-09

**Authors:** Rodrigo Yáñez-Sepúlveda, Vicente Javier Clemente-Suárez, Josivaldo de Souza-Lima, Gerson Ferrari, Juan Hurtado-Almonacid, Juan Pablo Zavala-Crichton, Claudio Hinojosa-Torres, Tomás Reyes-Amigo, Jacqueline Páez-Herrera, Guillermo Cortés-Roco, Pedro Valdivia-Moral, Jorge Olivares-Arancibia, Sandra Mahecha-Matsudo

**Affiliations:** a Universidad Andres Bello, Facultad de Educación y Ciencias Sociales, Viña del Mar, Chile; b Universidad Europea de Madrid, Faculty of Sports Sciences, Madrid, Spain; c Universidad de la Costa, Grupo de Investigación en Cultura, Educación y Sociedad, Barranquilla, Colombia; d Universidad Andres Bello, Facultad de Educación y Ciencias Sociales, Instituto del Deporte yBienestar, Las Condes, Chile; e Universidad Autónoma de Chile, Facultad de Ciencias de la Salud, Providencia, Chile; f Universidad de Santiago de Chile (USACH), Escuela de Ciencias de la Actividad Física, el Deporte y la Salud, Santiago, Chile; g Pontificia Universidad Católica de Valparaíso, Grupo EFIDAC, Valparaíso, Chile; h Universidad de Playa Ancha, Departamento de Ciencias de la Actividad Física, Observatorio de Ciencias de la Actividad Física (OCAF), Valparaíso, Chile; i Universidad Viña del Mar, Faculty Education, Viña del Mar, Chile; j Universidad de Granada, Facultad de Educación, Departamento de Didáctica de la Expresión Musical, Plástica y Corporal, Granada, Spain; k Universidad de las Américas, Facultad de Educación, Investigación en Actividad Física y Salud Escolar, Escuela de Pedagogía en Educación Física, Grupo AFySE, Santiago, Chile; l Universidad Mayor, Facultad de Medicina y Ciencias de la Salud, Santiago, Chile

**Keywords:** Exercise, Cardiorespiratory fitness, Health, Children

## Abstract

**Objective:**

To compare the cardiovascular risk and physical fitness, according to type of school in a national sample of Chilean school students.

**Methods:**

A total of 7,218 students participated, who completed all the national tests of the National System for Measuring the Quality of Education, which included physical fitness and anthropometric tests. The results were compared according to the type of educational establishment and anthropometric indicators were considered. Physical fitness was measured by lower extremity strength, abdominal strength, upper extremity strength, trunk flexibility, exertional heart rate, and cardiorespiratory fitness. Body mass index, heart rate, and waist-to-height ratio were analyzed as predictors of cardiovascular risk.

**Results:**

There were differences according to the type of establishment in the predictors of cardiovascular risk (p < 0.05). Differences were also found in the physical fitness tests evaluated (p < 0.01). Students in private schools (PSC) and subsidized schools (SC) had lower levels of cardiovascular risk and higher levels of physical fitness than public schools (PS) and schools with delegated administration (DA).

**Conclusions:**

In conclusion, students in educational establishments with a higher socioeconomic level have lower levels of cardiovascular risk and better physical fitness than students in public establishments. The authors suggest considering specific school interventions to mitigate cardiovascular risk and improve physical fitness among this vulnerable population. To this end, future studies should analyze the characteristics of physical activity and nutritional habits in schools to determine the factors that affect the results.

## Introduction

In Latin America, and specifically in Chile, educational institutions reflect significant socioeconomic and cultural disparities, with public and delegated administration schools primarily serving students from lower socioeconomic backgrounds, in contrast to subsidized and private schools that cater to those from higher economic levels.^1^ Despite educational principles advocating for universal accessibility and non-discrimination,^2^ Chilean schools are segregated by type, reinforcing socioeconomic divides. Physical activity (PA) levels are notably low, especially among students in public schools who exhibit higher physical inactivity and cardiovascular risk (CR) due to limited access to sports facilities compared to their counterparts in private institutions.^3^ This disparity is evident in physical fitness levels, with students, particularly females, in public or municipal schools performing worse than those in private schools,^4^ highlighting the influence of educational establishment type on physical fitness and CR.

The global epidemic of childhood obesity, with a significant impact on health that extends into adulthood, has seen a dramatic increase in prevalence among Chilean children, tripling to 23.9% by 2017.^5^ This surge in obesity is closely linked to adverse health outcomes, including metabolic syndromes like altered insulin levels, hypercholesterolemia,^6^ type 2 diabetes, and cardiovascular diseases from an early age.^7^ Early childhood obesity contributes to the development of cardiometabolic risk factors, such as dyslipidemia, diabetes, hypertension, and insulin resistance, which are significant predictors of atherosclerosis and subclinical cardiovascular diseases in later life.^8^ These findings underscore the urgent need for interventions targeting obesity and overweight in children to mitigate long-term cardiometabolic risks and improve overall health outcomes.

Research on Chilean children by Díez et al.^9^ demonstrates that BMI significantly mediates the link between muscular fitness and cardiometabolic risk, with normal-weight children having healthier profiles than overweight peers. Further studies indicate that children with low academic performance, malnutrition, and those from less affluent public schools face higher health risks.^4^ Physical activity (PA) levels are influenced by sociodemographic factors, such as age, gender, and socioeconomic status, showing that lower socioeconomic status is associated with reduced PA, which exacerbates health risks like obesity.^10^ Moreover, significant gender and socioeconomic disparities in PA levels have been found among adolescents in Global South Countries, including Chile, regardless of development or gender inequality indices, highlighting widespread disparities.^11^

In 2010, Chile introduced a fitness assessment in the national education survey, which led to a study by Garber et al.^12^ revealing significant geographic disparities in the physical fitness levels of eighth-grade students across Chile, influenced by geographic, educational, economic, and climatic factors.^12^ Building on this, the objective of the study was to compare the cardiovascular risk and physical fitness, according to type of school in a national sample of Chilean school students.

## Methods

### Study design

Comparative cross-sectional study. The objective of the SIMCE assessment is to evaluate the physical condition of schoolchildren. This evaluation is carried out within the educational establishments through the SIMCE-EFI program and consists of a nationally representative sampling among 8th grade students. Prior to these evaluations, the evaluators are trained by a team from the Chilean Ministry of Education, ensuring the correct execution of the project.^13^

### Participants

From a total of 30,654 students evaluated, 7,218 students participated in the national physical fitness tests (SIMCE-EF), with the study including all students who completed both the physical fitness and anthropometric assessments. The sample was collected based on stratified sampling according to the type of educational establishment, to quantify the number of participants, the total number of adolescents enrolled by region was considered.

### Cardiovascular risk

The waist-to-height ratio (WHtR) is determined by dividing the waist circumference (WC) by the height, serving as an effective index for assessing cardiovascular risk in children.^14^ Body Mass Index (BMI) is calculated by dividing weight in kilograms by the square of height in meters to evaluate the relationship between body mass and height. Anthropometric indicators include body weight, measured with the student on a scale in minimal clothing and barefoot, ensuring accuracy to one decimal place. Body height is assessed with the student standing straight, barefoot, and looking forward, with heels touching the base of the measuring rod, and recorded in centimeters. BMI was obtained by dividing weight by height squared (Weight (kg) /Height(m)^2^).^15^

Waist circumference is measured at the narrowest point between the lower rib and the iliac crest or at the midpoint between these two if the narrowest area is not apparent, recorded to one decimal place in centimeters.

### Physical fitness

The study incorporates various physical fitness assessments, detailed as follows:

• Abdominal Strength (Sit-ups): Participants perform crunches on a mat, aiming to touch a second mark 10 centimeters away with their middle fingers, within a minute to the rhythm of a sound stimulus. The total crunches are recorded.
• Cardiorespiratory Fitness (20 Meters Shuttle Run Test): Involves running between two lines 20 meters apart at increasing speeds with audio cues, concluding when the participant misses the cue twice. The total laps achieved within 15 minutes are documented.^16^
• Lower Limb Strength (Horizontal Jump Test): Participants jump as far as possible from a standstill, with two attempts allowed and the best distance in centimeters recorded.^13^
• Upper Limb Strength (Push-ups): Students perform push-ups, with males in a plank position and females using hands and knees for support, for 30 seconds. The total push-ups completed are tallied.^13^
• Sit and Reach Flexibility Test: Measures flexibility by having students stretch forward while seated with legs extended, recording the furthest distance reached over two attempts in centimeters.

### Type of school

For this study, the participant sample was categorized based on the type of school attended: Public School (PS), Subsidized with Shared Financing (SC), Delegated Administration School (DL), and Private School (PSC). The categories are defined by the Chilean Ministry of Education. In Chile, regardless of geographical location, schools are divided according to the type of funding as follows: public schools (PS), which are fully funded by the government, delegated administration schools (DA), which are funded through administration agreements signed with private law entities, subsidized schools (SC), which are funded jointly by the government and families, and private schools (PSC), which are fully funded by families.^17^

### Ethical aspects of the research

The study was conducted in accordance with the Declaration of Helsinki's ethical guideline, securing informed consent from both guardians and participants before conducting evaluations. It also received ethical approval from the bioethics and biosafety committee of the Pontificia Universidad Católica de Valparaíso (Code BIOPUCV-H516 2022), underlining its commitment to enhancing physical education quality.

### Statistical analysis

For the analysis of the study's findings, JAMOVI® version 2.3.21 for Windows® was employed. The mean and standard deviation statistics were used to describe the variables according to educational establishment. He first performed a normality test using the Kolmogorov and Smirnov test. The statistical approach encompassed descriptive statistics and an Analysis of Variance (ANOVA) test. Tukey's post hoc analysis, adjusted for sex and age, was utilized to compare metrics across different types of educational establishments. A significance threshold was set at p < 0.05, ensuring that observed differences were statistically meaningful.

## Results

Table 1 highlights variations in body composition and cardiovascular risk (CR) factors among students by school type, showing significant differences in Body Mass Index (BMI) and Waist-to-Height Ratio (WHtR) based on educational establishment. Despite similar average ages of around 15.9 years, variations in age distribution exist among school types. Height shows significant differences (p < .001), suggesting stature disparities, whereas weight differences are not statistically significant (p = 0.323). Notably, BMI and WHtR values significantly vary (p = 0.028 and p < .001, respectively), with private school students exhibiting lower averages, indicating potential disparities in obesity and overweight prevalence, and a healthier body composition concerning cardiovascular risk. These findings emphasize the impact of school type on students' health, particularly in relation to CR factors.

**Table 1:** Basic characteristics, body composition and CR in the group of students according to type of school.

*Variable*	PS (n = 883)	DA (n = 2011)	PSC (n = 417)	SC (n = 3907)	*p value*
**Age (years)**	15.9 ± 0.8	15.9 ± 0.8	15.9 ± 0.5	15.8 ± 0.7	< .001*
**Weight (kg)**	58.5 ± 12.3	58.6 ± 12.3	57.4 ± 10.6	58.5 ± 12.0	0.323
**Height (cm)**	161.2 ± 8.0	160.8 ± 8.1	162.3 ± 7.6	161.1 ± 8.0	< .001⁎⁎
**Waist (cm)**	73.4 ± 9.8	73.5 ± 10.0	72.0 ± 8.8	73.2 ± 10.2	0.095
**BMI**	18.1 ± 3.4	18.1 ± 3.4	17.6 ± 2.9	18.1 ± 3.4	0.028⁎⁎⁎
**WHtR**	0.456 ± 0.060	0.457 ± 0.060	0.444 ± 0.052	0.455 ± 0.062	< .001⁎⁎⁎⁎

PS, Public school; DA, Delegated administration; PSC, Private school; SC, Subsidized school.

⁎ Differences between PS with SC and DA with SC.

⁎⁎ Differences between PSC with all groups. Differences between DA and SC.

⁎⁎⁎ Differences between PSC with DA and PS.

⁎⁎⁎⁎ Differences between PSC with all groups. All differences with p-value < 0.05.

Table 2 demonstrates significant differences in physical fitness outcomes among students from various school types, highlighting the influence of educational environments on fitness levels. Private school students excel in sit-ups and horizontal jumps, reflecting superior core and lower limb strength (p < .001), whereas subsidized school students show the highest aerobic capacity, as indicated by VO2max values (p < .001). Trunk flexibility varies modestly across groups (p = 0.002), and private school students also lead in push-up performance (p < .001), signaling enhanced upper body strength.

**Table 2:** Variables of physical fitness in the group of students according to type of school.

*Variable*	PS (n = 883)	DA (n = 2011)	PSC (n = 417)	SC (n = 3907)	*p value*
**Sit ups (reps)**	22.4 ± 5.1	22.2 ± 5.3	23.8 ± 3.0	22.7 ± 4.8	< .001*
**VO_2max_ (mL/kg/min)**	28.5 ± 3.1	28.2 ± 1.8	28.7 ± 2.4	29.0 ± 3.6	< .001⁎⁎
**Trunk flexibility (cm)**	29.1 ± 8.1	29.6 ± 7.8	28.6 ± 9.4	29.3 ± 8.3	0.002⁎⁎⁎
**Horizontal jump (cm)**	143.5 ± 31.8	144.4 ± 35.6	159.3 ± 32.4	142.3 ± 34.8	< .001⁎⁎⁎⁎
**Push ups (reps)**	16.6 ± 9.1	15.4 ± 8.5	17.3 ± 8.8	15.3 ± 8.3	< .001⁎⁎⁎⁎⁎

PS, Public school; DA, Delegated administration; PSC, Private school; SSC, Subsidized school.

⁎ Differences between PSC and all groups. Differences between DA and SC.

⁎⁎ Differences between SC with PS and DA. Differences between PSC and DA. Differences between PS and DA. Differences between DA with PSC and SC.

⁎⁎⁎⁎ Differences between PSC with all groups.

⁎⁎⁎⁎⁎ Differences between PSC with DA and SC. Differences between PS with DA and SC.

⁎⁎⁎⁎⁎⁎⁎ Differences between PS with DA, PSC and SC. Differences between DA and SC.

**Figure 1:**
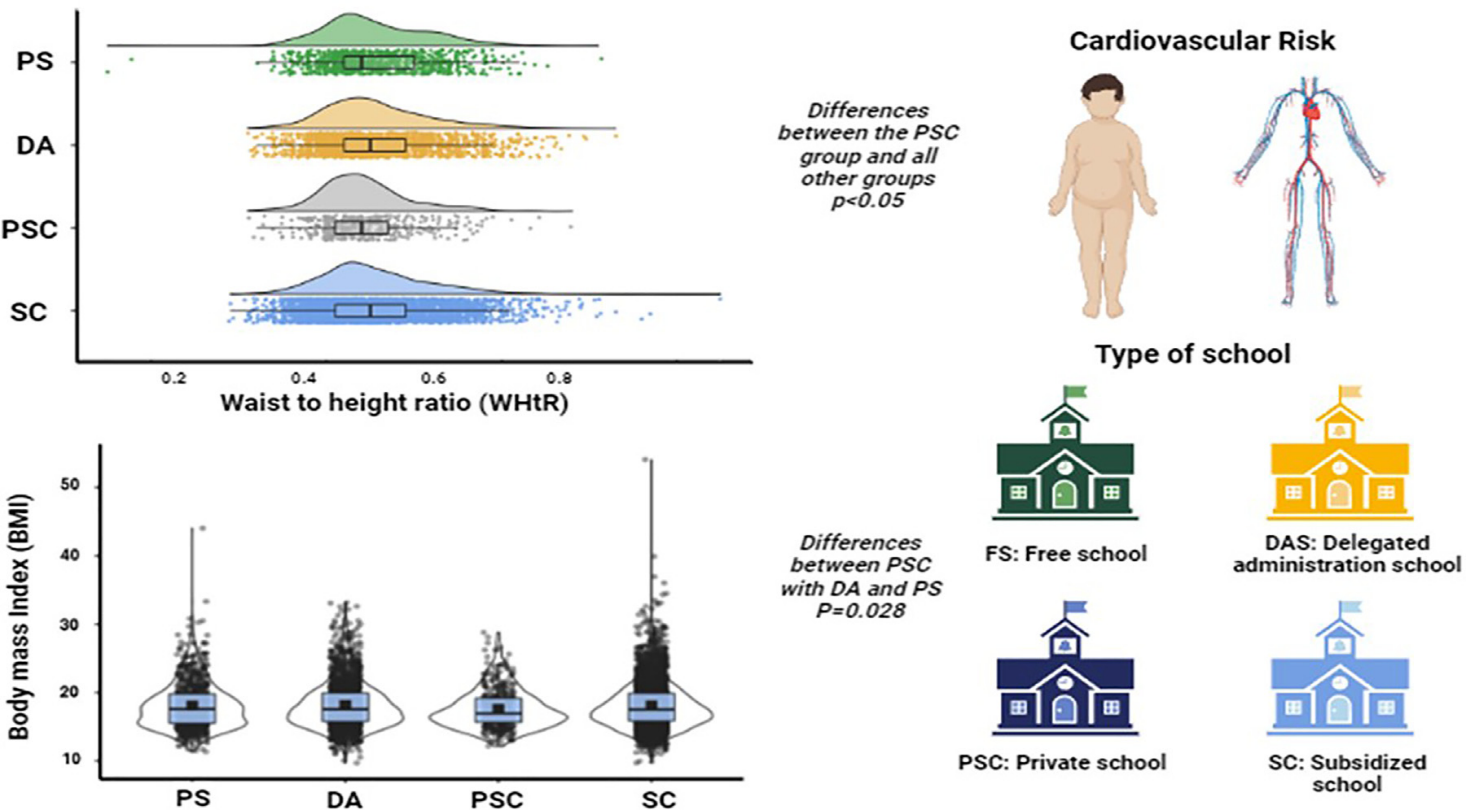
Waist-to-height ratio and body mass index according to type of school.

Figure 1 illustrates that students from Private Schools (PSC) and Subsidized Schools with Shared Financing (SSC) exhibit lower levels of cardiovascular risk (CR) when assessed through the waist-to-height ratio (p < 0.001) and Body Mass Index (BMI) (p = 0.026) compared to their counterparts in other educational groups. Differences were also observed between PSC with DA and PS (p = 0.028).

## Discussion

The primary aim of this investigation was to evaluate CR and physical fitness disparities based on gender, socioeconomic status, educational institution type, and geographical region within a comprehensive national cohort of Chilean students. The findings from this analysis revealed that the determinants of cardiorespiratory fitness exhibit significant variances contingent upon the physical fitness levels. In Chile, children of low socioeconomic status usually attend public schools and have few opportunities to engage in healthy behaviors. This may increase their risk of overweight/obesity and low muscular fitness. In this line, Suárez-Reyes et al.^18^ determined the association between the type of school they attend with markers related to overweight/obesity and the muscular condition of children in Chile. The associations were observed mainly in girls. Overall, girls who attended public schools showed an elevated risk of markers related to overweight/obesity and lower muscle fitness compared to those who attended charter or private schools. These results highlight the influence of social context and economic status on current and likely future child health. Even, an earlier study reported a prevalence of 40% overweight/obesity in Chilean children, with no differences according to socioeconomic level (i.e., poverty).^19^ Other studies also found significant disparities in nutritional status and muscle function among Chilean adolescents based on their vulnerability index. Castro et al.^20^ concluded that adolescents with higher vulnerability indices exhibit poorer nutritional and muscular function indicators. These findings underscore the need for targeted interventions to enhance health and physical performance within this vulnerable group. The results align with existing literature indicating that socioeconomic factors profoundly influence children's health outcomes and physical capabilities, suggesting comprehensive strategies are required to address these inequalities effectively.

Moreover, regional comparisons of Body Mass Index (BMI), and Waist-to-Hip Ratio (WHR) disclosed notable differences. Pertaining to the variances in cardiorespiratory fitness determinants relative to fitness levels, prior research has corroborated that physical fitness serves as a formidable prognosticator of imminent health outcomes and various comorbid conditions.^21^

Substantial evidence^22^ has underscored a negative association between BMI and aerobic capacity, denoting that an escalation in BMI inversely affects cardiorespiratory fitness scores. This pattern, consistent among adults sharing similar traits, underscores the dire health implications should these trends persist unaltered. Lópes et al.^23^ elucidated that an enhanced cardiovascular profile is intrinsically linked to superior aerobic capacity. Concurrently, Nqweniso et al.^24^ highlighted that a demographic of boys and girls characterized by diminished levels of light physical activity (PA) and lower Vo2 max scores exhibited a higher prevalence of total and high-density lipoprotein cholesterol with increasing age. This condition suggests a diminished likelihood among youth to achieve the recommended PA guidelines. Additionally, it posits that physical fitness, particularly cardiorespiratory fitness, may mitigate the adverse effects of BMI on cardiovascular disease risk and the prevalence of obesity-related comorbidities, notably among children and adolescents.^25^

Conversely, a negative correlation has been established between adipose tissue accumulation and cardiorespiratory fitness in children classified as obese compared to their normal-weight counterparts, a phenomenon observed irrespective of their physical activity (PA) levels.^26^ In a similar vein, research by Olagbegi et al.^23^ has pinpointed that children experiencing the highest incidence of cardiovascular events are those with elevated Human Development Index (HDI) scores and higher systolic blood pressure readings. Additionally, an investigation conducted by Delgado et al.^7^ on Chilean schoolchildren revealed that those of normal weight exhibited superior outcomes in health-related physical fitness metrics compared to their overweight and obese peers. This finding is consistent with the research conducted by Ceschia et al.,^27^ which demonstrated that overweight and obese children possess significantly reduced levels of physical fitness in contrast to their normal-weight counterparts. This underscores an observable trend where physical fitness levels are inversely impacted by weight status, as corroborated by various studies.^9^

Chen et al.^28^ and Lopes et al.^23^ studies reveal that in Chinese and Brazilian children and adolescents, physical fitness peaks within a normative BMI range, with both lower and higher BMI extremes linked to reduced fitness, suggesting an inverted U-shaped correlation across age groups. These findings highlight the importance of maintaining an optimal BMI to enhance physical fitness and mitigate disease risk, emphasizing a balanced approach involving regular physical activity and dietary moderation to keep BMI within recommended levels. This underscores the complex relationship between BMI and physical fitness, advocating for health and wellness strategies that promote physical activity and dietary balance to improve overall physical fitness and health outcomes.

In analyzing the impact of waist circumference differences by gender and the administrative classification of educational institutions among participants, the present study found no statistically significant disparities. However, an interesting pattern emerged, indicating that students from private schools exhibited lower waist circumferences compared to their peers in public, delegated administration, and subsidized educational settings. This observation suggests a potential link between socioeconomic status, as inferred from the type of educational institution attended, and health outcomes related to obesity and metabolic health. Further exploration of this phenomenon is supported by Smith et al.,^29^ who found that children and adolescents attending schools in more affluent areas had better access to resources that promote a healthier lifestyle, such as quality physical education, healthier food options, and safer environments for active play, contributing to healthier body metrics. Similarly, Johnson et al.^30^ demonstrated that socioeconomic factors play a significant role in determining health outcomes, including obesity rates among children, suggesting that interventions to reduce waist circumference should not only focus on individual behaviors but also address broader socioeconomic and environmental determinants.

Recent studies highlight a negative correlation between waist circumference (an indicator of adiposity) and physical fitness in Chilean children and adolescents, showing that increased body fat adversely affects lower extremity strength and overall physical performance.^31^ Further research by Bustos et al.^32^ supports this, finding that children with healthier lifestyles exhibit lower BMI and waist circumference, alongside better cardiorespiratory fitness and strength. This relationship is partly due to declining physical activity levels, increased sedentary behavior, and rising abdominal obesity among this population.^33^ These findings underscore the importance of interventions to promote physical activity and reduce sedentary habits, aiming to improve physical fitness and counteract the negative health impacts of adiposity in children and adolescents.

Urban living significantly impacts children's health, making them particularly vulnerable to its adverse effects due to their limited autonomy and the influence of their environment. The expansion of urban areas introduces environmental stressors like increased air and noise pollution and a lack of green spaces, leading to more sedentary lifestyles and an energy imbalance. This contributes to higher rates of overweight and obesity among urban children, adversely affecting their cardiovascular health.^34^ A study by Islam et al.^35^ comparing cardiovascular disease risk factors in urban versus rural schoolchildren in Bangladesh found a significant urban-rural gradient in obesity prevalence, with urban children showing higher rates of obesity and overweight. This underscores the critical need to address urban-specific health risks and implement strategies to mitigate the impact of urban environments on children's cardiovascular well-being.

Monitoring physical fitness and body weight in children and adolescents is crucial for preventing obesity and related health issues, with the prepubertal phase being particularly vital for establishing lasting healthy habits. Schools are identified as key venues for health interventions and monitoring due to their access to diverse groups of children. This study highlights the need for region-specific programs that consider local socioeconomic and demographic factors, given the observed variation in health risk factors across different parts of the country. Additionally, there is a noted research gap regarding the impact of sociodemographic variables on health risks like arterial hypertension in children. Joubert et al.^36^ emphasize the importance of further research to understand and address health disparities influenced by sociodemographic differences, stressing the importance of tailored interventions to improve health outcomes in the pediatric population.

## Conclusion

In conclusion, students in educational establishments with a higher socioeconomic level have lower levels of cardiovascular risk and better physical fitness than students in public establishments. The authors suggest considering specific school interventions to mitigate cardiovascular risk and improve physical fitness among this vulnerable population. To this end, future studies should analyze the characteristics of physical activity and nutritional habits in schools to determine the factors that affect the results.

## Conflicts of interest

The authors declare no conflicts of interest.
